# Targeting inflammation with collagen

**DOI:** 10.1002/ctm2.831

**Published:** 2022-05-23

**Authors:** Dennis Schwarz, Marie Lipoldová, Holger Reinecke, Yahya Sohrabi

**Affiliations:** ^1^ Department of Cardiology I ‐ Coronary and Peripheral Vascular Disease, Heart Failure University Hospital Münster Münster Germany; ^2^ Laboratory of Signal Transduction Institutes of Molecular Genetics of the Czech Academy of Sciences Prague Czechia

**Keywords:** collagen, COVID‐19, ECM, inflammation, MMPs

## Abstract

Tissue damage caused by an infection oran autoimmune disease triggers degradation of collagen in the extracellular matrix (ECM), which further enhances inflammation. Therefore, improving ECM in aninflamed tissue can be exploited as a potential therapeutic target. A recentstudy emphasised an innovative approach against COVID‐19 using polymerised type I collagen (PTIC) that improves disease severity through a hitherto unknownmechanism. In this paper, we provide an overview of potential mechanism thatmay explain the anti‐inflammatory effect of collagen peptides. In addition,the paper includes a brief summary of possible side effect of collagendeposition in inflammatory diseases. Altogether, current knowledge suggeststhat collagen may potentially reduce the residual risk in inflammatorydiseases; however, the detailed mechanism remains elusive.

## 1 THE EXTRACELLULAR MATRIX

The extracellular matrix (ECM) is a three‐dimensional meshwork composed of different macromolecules, predominantly collagens that form a bioactive scaffold providing structural and biochemical support to cells. It is a highly dynamic yet strictly regulated tissue component and the balance between its synthesis and degradation is essential for tissue architecture, physiological homeostasis and repair.^1^ ECM synthesis is regulated by cytokines such as transforming growth factor‐β (TGF‐β), while ECM turnover is orchestrated by matrix metalloproteinases (MMPs) as well as tissue inhibitors of metalloproteinases (TIMPs).^1^ Many pathological conditions arise from dysregulated ECM remodelling. Thus, modulation of collagen turnover in the ECM could serve as a therapeutic target.

## 2 ECM REMODELING AND INFLAMMATION

**FIGURE 1:**
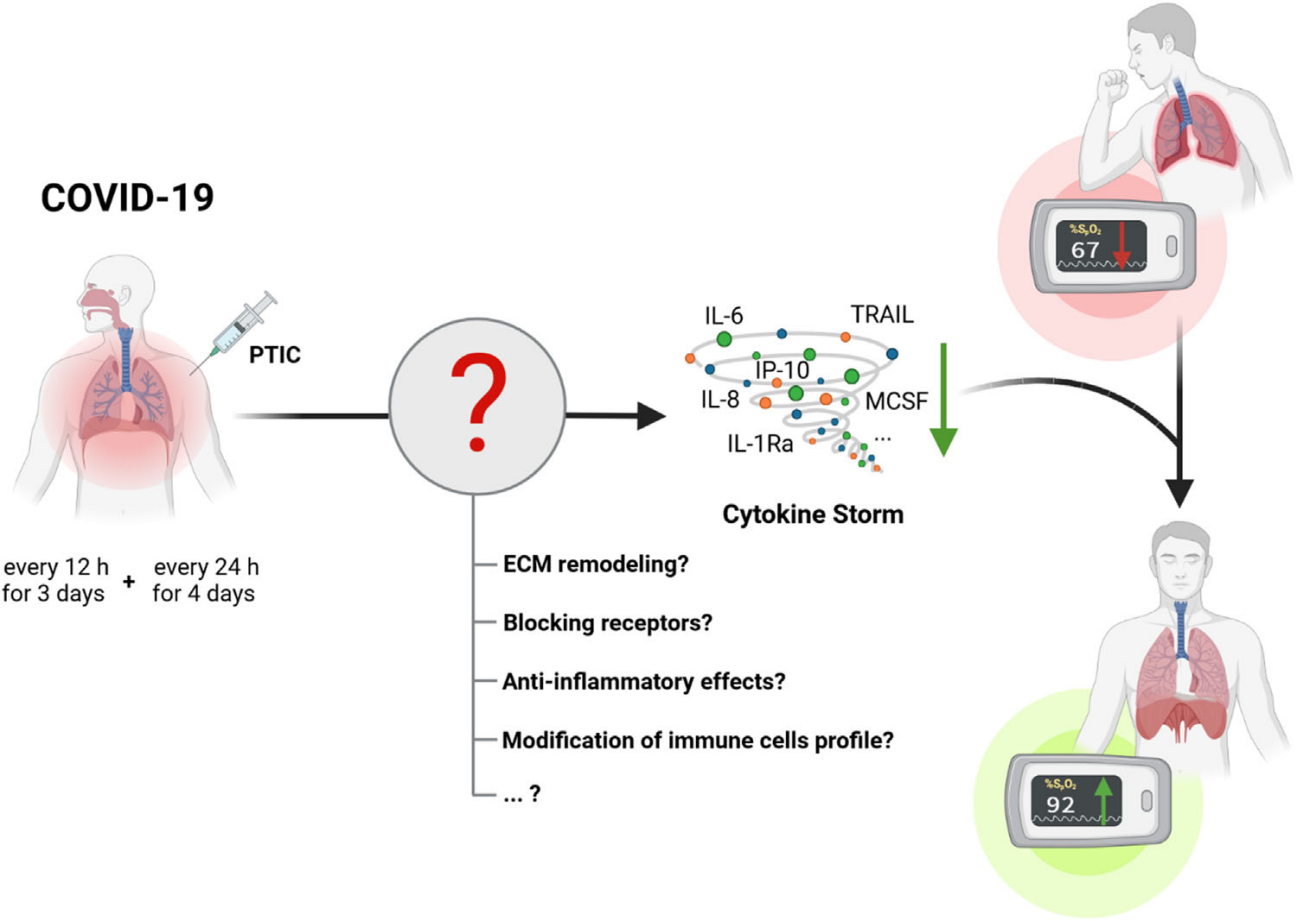
Polymerised type I collagen (PTIC) exhibits immunomodulatory effects in COVID‐19. The PTIC‐treated group showed significant improvement in disease severity. Likewise, the levels of inflammatory cytokines dramatically decreased and the mean oxygen saturation rates improved to > 92%

A recent randomised, placebo‐controlled clinical trial demonstrated an innovative approach of treating symptomatic outpatient COVID‐19 infection by intramuscular injection of polymerised type I collagen (PTIC).^2^ It was shown that PTIC has immunomodulatory properties and reduces the excessive release of inflammatory mediators arising due to cytokine release syndrome, which determines the clinical course, especially in the second week of infection. One day after completing the treatment, the patients in the treatment group presented a significant increase in mean oxygen saturation readings with values of 92% or higher as well as a shorter duration of symptoms (Figure 1).^2^ The present study delivers a novel approach of targeting systemic inflammation by administration of an ECM component. However, the underlying mechanism of collagen‐induced immunomodulation remains elusive.

It is known that there is a close interplay between the ECM and both adaptive and innate immunity, as collagen deposition and degradation are closely related to the activity of immune cells. On the one hand, MMPs produced by activated immune cells such as monocytes/macrophages promote the migration of immune cells, thereby enhancing MMP production and inflammation. On the other hand, degraded collagen itself acts as a strong chemoattractant for immune cells.^1^ Collagen deposition can mediate both normal and dysregulated tissue repair after infection. Pathological accumulation of excess ECM defined as fibrosis was observed in many chronic infectious diseases caused by microbial agents such as bacteria, viruses such as HIV,^3^ influenza and SARS‐CoV‐2^4^ and parasites such as *Leishmania*.^5^ In HIV infection, collagen deposition can lead to a changed structure of lymphatic tissues, thereby decreasing the count of CD4^+^ and CD8^+^ T cells and impairing antigen‐lymphocyte interactions.^3^

Furthermore, collagen type I deposition and the fibrosis score were significantly more prominent in the lungs of COVID‐19 patients. This patient group also presented more pronounced infiltration of the lungs by different immune cell types, such as macrophages and neutrophils.^4^ Overdeposition of collagen in COVID‐19 infection can lead to pulmonary fibrosis, which is also a characteristic feature in other diseases, such as idiopathic pulmonary fibrosis or late‐stage bronchial asthma, where anti‐inflammatory therapies have already been established for a long time. The synthesis of both ECM proteins and MMPs is significantly increased in idiopathic pulmonary fibrosis, with the result that in addition to the general inflammation parameter C‐reactive protein (CRP), increased serum concentrations of ECM degraded fragments and MMPs are correlated with the progression of the disease.^6^

## 3 IMMUNOMODULATORY EFFECT OF COLLAGEN

**FIGURE 2:**
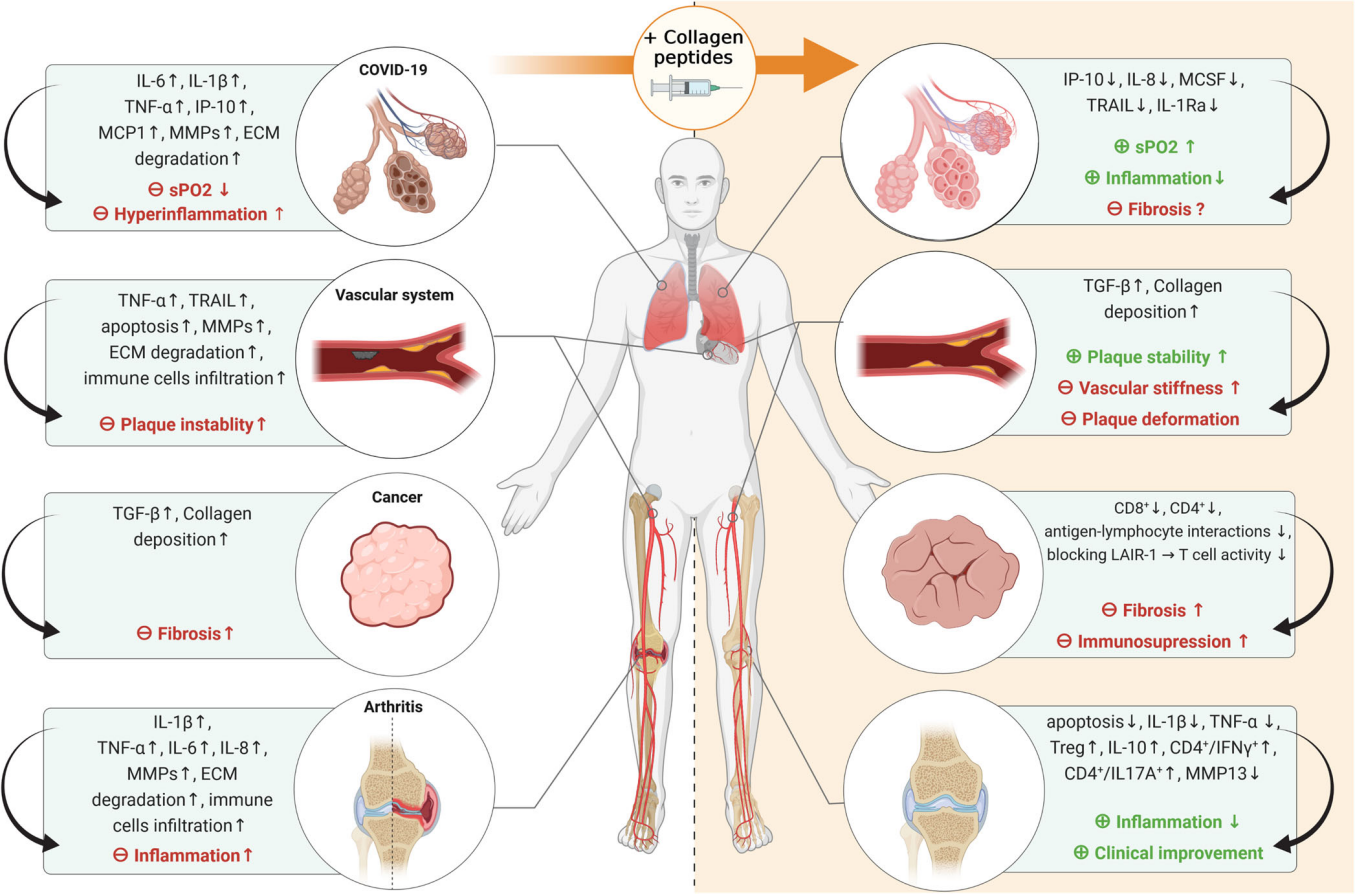
Potential immunomodulatory mechanisms and consequences of collagen deposition or administration of collagen peptides

Intra‐articular administration of PTIC in patients with symptomatic knee osteoarthritis (OA) not only led to a significant clinical improvement but also to a significant reduction in the erythrocyte sedimentation rate (ESR) as well as to a significant reduction in interleukin (IL)‐1β and tumour necrosis factor (TNF)‐α‐expressing peripheral cells compared to placebo. Furthermore, the number of regulatory T cells (T_regs_) and IL‐10‐expressing peripheral cells was increased after intervention.^7^ Additionally, it could be shown in a mouse model of rheumatoid arthritis that joint regeneration after PTIC treatment was accompanied by a decrease in CD4^+^/IL17A^+^ T‐cell number and an increase in T_regs_ and CD4^+^/IFN‐γ^+^ T cells.^7^ Interestingly, oral supplementation with hydrolysed collagen also led to an improvement in OA by inducing collagen synthesis, while reduced MMP13 production and reduced apoptosis could be observed.^8^ Reduced collagen degradation and its immunosuppressive effect also seems to be the key element in lowering cytokine release in COVID‐19 infection (Figure 2).

Admittedly, the potential of collagen to reduce its own degradation by inhibition of MMPs is beneficial because degradation of collagen in atherosclerotic plaques leads to plaque instability.^9^ On the other hand, overdeposition of collagen in the vessel wall as well as fragmentation of elastic fibers can accelerate vascular stiffness and repetitive plaque deformation.^9^ Moreover, in fibrosis, inflammatory T cells and dendritic cells migrate towards chemoattractants along collagen fibers. However, as tissue infiltration of these cells is nonproteolytic, they are not able to migrate through tissue‐associated dense collagen meshworks as produced by tumors. Furthermore, collagen‐rich ECM leads to the appearance of regulatory tumor‐associated macrophages, which suppress the activity of T cells and thereby limit the response to immunotherapy.^10^ By inhibiting collagen crosslinking, an important step during fibrillogenesis, fibrosis and stromal stiffening can be attenuated, thereby offering a new therapeutic approach.^10^ Nevertheless, collagen can also directly suppress T‐cell activity by binding to leukocyte‐associated Ig‐like receptor‐1 (LAIR‐1), an immune‐inhibitory transmembrane receptor found on most peripheral blood mononuclear cells (PBMCs) transmitting an immunosuppressive effect^10^ (Figure 2).

## 4 PERSPECTIVE AND CONCLUSION

It is well accepted that despite the beneficial effects of collagen, there are also potential detrimental effects due to its immunomodulatory capacities. In the current study, as the patients received PTIC, it is still uncertain whether the patients present any potential side effects, such as developing fibrosis in other tissues over time. Therefore, long‐term follow‐up should be considered. Furthermore, it will be interesting to check if the clinical course of the COVID‐19 infection or the time of initiating the treatment influences the outcome. Concentrations of MMPs and TIMPs (MMP/TIMP ratio) and collagen fragments as well as the number of infiltrating immune cells in the circulation or bronchial fluid could be biomarkers to assess the progression of the disease and may provide evidence for the mechanism of PTIC action against COVID‐19. Detailed information on the mechanism will help prevent potential side effects after systemic injection of collagen compounds.

Altogether, the current knowledge sheds light on the role of collagen in inflammatory disease development and opens up new perspectives by using collagen as an innovative anti‐inflammatory therapeutic target, yet the mechanism and potential side effects should be carefully investigated.

## CONFLICT OF INTEREST

The authors declare no conflict of interest.
